# Supplementary material for: End Sequence Analysis Toolkit (ESAT) expands the extractable information from single-cell RNA-seq data
Source: Genome Res. 2016 Oct;26(10):1397–410. doi: 10.1101/gr.207902.116 (PMC5052061; doi:10.1101/gr.207902.116)
Supplement: Supplemental Material [file supp_gr.207902.116_Supp_Materials.tar.gz › ESAT-esat_v0.1/doc/html/classumms_1_1core_1_1annotation_1_1_annotation_list_3_01_t_01extends_01_annotation_01_4.html]

ESAT: umms.core.annotation.AnnotationList< T extends Annotation > Class Reference


ESAT


- Main Page
- Related Pages
- Packages
- Classes
- Files

- Class List
- Class Index
- Class Hierarchy
- Class Members

All Classes Namespaces Files Functions Variables Enumerator Pages

- umms
- core
- annotation
- AnnotationList< T extends Annotation >

Public Member Functions |
Static Public Attributes |
Protected Attributes |
List of all members

umms.core.annotation.AnnotationList< T extends Annotation > Class Reference

Inheritance diagram for umms.core.annotation.AnnotationList< T extends Annotation >:

[legend]

Collaboration diagram for umms.core.annotation.AnnotationList< T extends Annotation >:

[legend]

|  |  |
| --- | --- |
| Public Member Functions | |
|  | AnnotationList () |
|  | |
|  | AnnotationList (CoordinateSpace cs) |
|  | |
|  | AnnotationList (CoordinateSpace cs, Collection<?extends T > annotations) |
|  | |
|  | AnnotationList (CoordinateSpace cs, T...annotations) |
|  | |
| int | size () |
|  | |
| double | getGlobalCount () |
|  | |
| double | getCount (Annotation region, boolean fullyContained) |
|  | |
| double | getCountExcludingRegion (Annotation region, Annotation excluded) |
|  | |
| int | getBasesCovered (Annotation region, boolean fullyContained) |
|  | |
| CloseableIterator< T > | getOverlappingAnnotations (Annotation region, boolean fullyContained) |
|  | |
| int | getNumOverlappingAnnotations (Annotation region) |
|  | |
| CloseableIterator< T > | getPermutedAnnotations (Annotation region) |
|  | |
| CloseableIterator< T > | iterator () |
|  | |
| void | addFilter (Predicate< T > filter) |
|  | |
| void | addFilters (Collection< Predicate< T >> filters) |
|  | |
| void | addAll (Iterable<?extends T > annotations) |
|  | |
| void | addAll (AnnotationList<?extends T > otherSet) |
|  | |
| void | add (T annotation) |
|  | |
| void | removeAnnotation (T annotation) |
|  | |
| void | collapse () |
|  | |
| AnnotationList< T > | intersect (AnnotationList<?extends Annotation > other) |
|  | |
| AnnotationList< T > | minus (AnnotationList<?extends Annotation > other) |
|  | |
| AnnotationList< T > | getOverlappingAnnotationList (AnnotationList<?extends Annotation > other) |
|  | |
| boolean | hasAnnotationThatOverlaps (Annotation region) |
|  | |
| boolean | hasAnnotationThatContains (Annotation region) |
|  | |
| AnnotationList< T > | getOverlappingAnnotationList (AnnotationList<?extends Annotation > other, boolean fullyContained) |
|  | |
| T | getClosest (final Annotation query) |
|  | |
| T | getClosestNonOverlapping (final Annotation query) |
|  | |
| T | getClosest (final Annotation query, boolean nonOverlapping) |
|  | |
| T | getClosestUpstream (final Annotation query) |
|  | |
| T | getClosestDownstream (final Annotation query) |
|  | |
| Integer | getDistanceToClosest (final Annotation query) |
|  | |
| List< T > | toList () |
|  | |
| CoordinateSpace | getCoordinateSpace () |
|  | |
| double | getRefSequenceLambda (String refname) |
|  | |
| double | getCountStrandedExcludingRegion (Annotation region, Annotation excluded) |
|  | |

|  |  |
| --- | --- |
| Static Public Attributes | |
| static boolean | ENFORCE\_REFERENCE = false |
|  | |

|  |  |
| --- | --- |
| Protected Attributes | |
| final CoordinateSpace | coordinateSpace |
|  | |
| TreeMap< String, IntervalTree  < T > > | annotations = new TreeMap<String, IntervalTree<T>>() |
|  | |

## Detailed Description

Author
:   engreitz Class for storing and oeprating on a set of annotations in memory Allows multiple identical annotations. Iterators, counting, etc. always consider the filters present in the AnnotationList. TODO: Add a different class that will dynamically read BED files, perhaps by using an indexing system TODO: Add a global extension factor TODO: Integrate with modified reader classes that can populate an AnnotationList from a file (this class should not be responsible for parsing or reading - only for storing and manipulating) TODO: Alternative (faster?) implementation: remove all annotations that don't pass filters so you don't have to test every annotation every time you iterate. In this implementation, addFilter would apply the filter to all annotations upon addition, removing those that do not pass.

Parameters
:   |  |  |
    | --- | --- |
    | <T> |  |

## Constructor & Destructor Documentation

|  |  |  |  |  |
| --- | --- | --- | --- | --- |
| umms.core.annotation.AnnotationList< T extends Annotation >.AnnotationList | ( |  | ) |  |

|  |  |  |  |  |  |
| --- | --- | --- | --- | --- | --- |
| umms.core.annotation.AnnotationList< T extends Annotation >.AnnotationList | ( | CoordinateSpace | *cs* | ) |  |

Initializing with a coordinate space allows for some additional filtering and sanity checks

Parameters
:   |  |  |
    | --- | --- |
    | cs |  |

|  |  |  |  |
| --- | --- | --- | --- |
| umms.core.annotation.AnnotationList< T extends Annotation >.AnnotationList | ( | CoordinateSpace | *cs*, |
|  |  | Collection<?extends T > | *annotations* |
|  | ) |  |  |

|  |  |  |  |
| --- | --- | --- | --- |
| umms.core.annotation.AnnotationList< T extends Annotation >.AnnotationList | ( | CoordinateSpace | *cs*, |
|  |  | T... | *annotations* |
|  | ) |  |  |

## Member Function Documentation

|  |  |  |  |  |  |
| --- | --- | --- | --- | --- | --- |
| void umms.core.annotation.AnnotationList< T extends Annotation >.add | ( | T | *annotation* | ) |  |

Add an annotation

Parameters
:   |  |  |
    | --- | --- |
    | annotation |  |

|  |  |  |  |  |  |
| --- | --- | --- | --- | --- | --- |
| void umms.core.annotation.AnnotationList< T extends Annotation >.addAll | ( | Iterable<?extends T > | *annotations* | ) |  |

Add a collection of annotations

Parameters
:   |  |  |
    | --- | --- |
    | annotations |  |

|  |  |  |  |  |  |
| --- | --- | --- | --- | --- | --- |
| void umms.core.annotation.AnnotationList< T extends Annotation >.addAll | ( | AnnotationList<?extends T > | *otherSet* | ) |  |

Add another AnnotationList. Duplicates allowed

Parameters
:   |  |  |
    | --- | --- |
    | otherSet |  |

|  |  |  |  |  |  |
| --- | --- | --- | --- | --- | --- |
| void umms.core.annotation.AnnotationList< T extends Annotation >.addFilter | ( | Predicate< T > | *filter* | ) |  |

|  |  |  |  |  |  |
| --- | --- | --- | --- | --- | --- |
| void umms.core.annotation.AnnotationList< T extends Annotation >.addFilters | ( | Collection< Predicate< T >> | *filters* | ) |  |

|  |  |  |  |  |
| --- | --- | --- | --- | --- |
| void umms.core.annotation.AnnotationList< T extends Annotation >.collapse | ( |  | ) |  |

Collapse overlapping annotations.

|  |  |  |  |
| --- | --- | --- | --- |
| int umms.core.annotation.AnnotationList< T extends Annotation >.getBasesCovered | ( | Annotation | *region*, |
|  |  | boolean | *fullyContained* |
|  | ) |  |  |

|  |  |  |  |  |  |
| --- | --- | --- | --- | --- | --- |
| T umms.core.annotation.AnnotationList< T extends Annotation >.getClosest | ( | final Annotation | *query* | ) |  |

Parameters
:   |  |  |
    | --- | --- |
    | query |  |

Returns
:   Closest annotation in the set, or null if there are no annotations on the same chromosome

|  |  |  |  |
| --- | --- | --- | --- |
| T umms.core.annotation.AnnotationList< T extends Annotation >.getClosest | ( | final Annotation | *query*, |
|  |  | boolean | *nonOverlapping* |
|  | ) |  |  |

Parameters
:   |  |  |
    | --- | --- |
    | query |  |

Returns
:   Closest (non-overlapping) annotation in the set, or null if there are no matches on the same chromosome

|  |  |  |  |  |  |
| --- | --- | --- | --- | --- | --- |
| T umms.core.annotation.AnnotationList< T extends Annotation >.getClosestDownstream | ( | final Annotation | *query* | ) |  |

Returns the closest non-overlapping annotation 3-prime of the query, considering strand.

Parameters
:   |  |  |
    | --- | --- |
    | query |  |

Returns

|  |  |  |  |  |  |
| --- | --- | --- | --- | --- | --- |
| T umms.core.annotation.AnnotationList< T extends Annotation >.getClosestNonOverlapping | ( | final Annotation | *query* | ) |  |

Parameters
:   |  |  |
    | --- | --- |
    | query |  |

Returns
:   Closest non-overlapping annotation in the set, or null if there are no non-overlapping annotations on the same chromosome

|  |  |  |  |  |  |
| --- | --- | --- | --- | --- | --- |
| T umms.core.annotation.AnnotationList< T extends Annotation >.getClosestUpstream | ( | final Annotation | *query* | ) |  |

Returns the closest non-overlapping annotation 5-prime of the query, considering strand.

Parameters
:   |  |  |
    | --- | --- |
    | query |  |

Returns

|  |  |  |  |  |
| --- | --- | --- | --- | --- |
| CoordinateSpace umms.core.annotation.AnnotationList< T extends Annotation >.getCoordinateSpace | ( |  | ) |  |

|  |  |  |  |
| --- | --- | --- | --- |
| double umms.core.annotation.AnnotationList< T extends Annotation >.getCount | ( | Annotation | *region*, |
|  |  | boolean | *fullyContained* |
|  | ) |  |  |

|  |  |  |  |
| --- | --- | --- | --- |
| double umms.core.annotation.AnnotationList< T extends Annotation >.getCountExcludingRegion | ( | Annotation | *region*, |
|  |  | Annotation | *excluded* |
|  | ) |  |  |

|  |  |  |  |
| --- | --- | --- | --- |
| double umms.core.annotation.AnnotationList< T extends Annotation >.getCountStrandedExcludingRegion | ( | Annotation | *region*, |
|  |  | Annotation | *excluded* |
|  | ) |  |  |

|  |  |  |  |  |  |
| --- | --- | --- | --- | --- | --- |
| Integer umms.core.annotation.AnnotationList< T extends Annotation >.getDistanceToClosest | ( | final Annotation | *query* | ) |  |

Parameters
:   |  |  |
    | --- | --- |
    | query |  |

Returns
:   distance in coordinate space between the query and the closest element in the set, or null if there are no others on the same chromosome

|  |  |  |  |  |
| --- | --- | --- | --- | --- |
| double umms.core.annotation.AnnotationList< T extends Annotation >.getGlobalCount | ( |  | ) |  |

|  |  |  |  |  |  |
| --- | --- | --- | --- | --- | --- |
| int umms.core.annotation.AnnotationList< T extends Annotation >.getNumOverlappingAnnotations | ( | Annotation | *region* | ) |  |

|  |  |  |  |  |  |
| --- | --- | --- | --- | --- | --- |
| AnnotationList<T> umms.core.annotation.AnnotationList< T extends Annotation >.getOverlappingAnnotationList | ( | AnnotationList<?extends Annotation > | *other* | ) |  |

Parameters
:   |  |  |
    | --- | --- |
    | other |  |

Returns
:   AnnotationList containing all annotations that overlap an annotation in other.

|  |  |  |  |
| --- | --- | --- | --- |
| AnnotationList<T> umms.core.annotation.AnnotationList< T extends Annotation >.getOverlappingAnnotationList | ( | AnnotationList<?extends Annotation > | *other*, |
|  |  | boolean | *fullyContained* |
|  | ) |  |  |

Parameters
:   |  |  |
    | --- | --- |
    | other |  |
    | fullyContained |  |

Returns
:   AnnotationList containing all annotations that overlap (or contained by) an annotation in other.

|  |  |  |  |
| --- | --- | --- | --- |
| CloseableIterator<T> umms.core.annotation.AnnotationList< T extends Annotation >.getOverlappingAnnotations | ( | Annotation | *region*, |
|  |  | boolean | *fullyContained* |
|  | ) |  |  |

Here is the call graph for this function:

|  |  |  |  |  |  |
| --- | --- | --- | --- | --- | --- |
| CloseableIterator<T> umms.core.annotation.AnnotationList< T extends Annotation >.getPermutedAnnotations | ( | Annotation | *region* | ) |  |

|  |  |  |  |  |  |
| --- | --- | --- | --- | --- | --- |
| double umms.core.annotation.AnnotationList< T extends Annotation >.getRefSequenceLambda | ( | String | *refname* | ) |  |

|  |  |  |  |  |  |
| --- | --- | --- | --- | --- | --- |
| boolean umms.core.annotation.AnnotationList< T extends Annotation >.hasAnnotationThatContains | ( | Annotation | *region* | ) |  |

Parameters
:   |  |  |
    | --- | --- |
    | region |  |

Returns
:   true if this has any Annotations that fully contain region

|  |  |  |  |  |  |
| --- | --- | --- | --- | --- | --- |
| boolean umms.core.annotation.AnnotationList< T extends Annotation >.hasAnnotationThatOverlaps | ( | Annotation | *region* | ) |  |

Parameters
:   |  |  |
    | --- | --- |
    | region |  |

Returns
:   true if this has any Annotations that overlap region

|  |  |  |  |  |  |
| --- | --- | --- | --- | --- | --- |
| AnnotationList<T> umms.core.annotation.AnnotationList< T extends Annotation >.intersect | ( | AnnotationList<?extends Annotation > | *other* | ) |  |

Returns the intersection of this AnnotationList with another. Behaves like bedIntersect Different than getOverlappers

Parameters
:   |  |  |
    | --- | --- |
    | other |  |

Returns

|  |  |  |  |  |
| --- | --- | --- | --- | --- |
| CloseableIterator<T> umms.core.annotation.AnnotationList< T extends Annotation >.iterator | ( |  | ) |  |

|  |  |  |  |  |  |
| --- | --- | --- | --- | --- | --- |
| AnnotationList<T> umms.core.annotation.AnnotationList< T extends Annotation >.minus | ( | AnnotationList<?extends Annotation > | *other* | ) |  |

Returns a new AnnotationList that does not contain any annotations that overlap annotations in other

Parameters
:   |  |  |
    | --- | --- |
    | other |  |

Returns

|  |  |  |  |  |  |
| --- | --- | --- | --- | --- | --- |
| void umms.core.annotation.AnnotationList< T extends Annotation >.removeAnnotation | ( | T | *annotation* | ) |  |

|  |  |  |  |  |
| --- | --- | --- | --- | --- |
| int umms.core.annotation.AnnotationList< T extends Annotation >.size | ( |  | ) |  |

|  |  |  |  |  |
| --- | --- | --- | --- | --- |
| List<T> umms.core.annotation.AnnotationList< T extends Annotation >.toList | ( |  | ) |  |

Returns
:   A list of all annotations passing the filter

## Member Data Documentation

TreeMap<String, IntervalTree<T> > umms.core.annotation.AnnotationList< T extends Annotation >.annotations = new TreeMap<String, IntervalTree<T>>()

protected

final CoordinateSpace umms.core.annotation.AnnotationList< T extends Annotation >.coordinateSpace

protected

boolean umms.core.annotation.AnnotationList< T extends Annotation >.ENFORCE\_REFERENCE = false

static

---

The documentation for this class was generated from the following file:

- java/umms/core/annotation/AnnotationList.java


---

Generated on Fri Aug 15 2014 15:11:08 for ESAT by  

 1.8.7
